# Sapitinib Reverses Anticancer Drug Resistance in Colon Cancer Cells Overexpressing the ABCB1 Transporter

**DOI:** 10.3389/fonc.2020.574861

**Published:** 2020-10-09

**Authors:** Hai-Ling Gao, Pranav Gupta, Qingbin Cui, Yunali V. Ashar, Zhuo-Xun Wu, Leli Zeng, Zi-Ning Lei, Qiu-Xu Teng, Charles R. Ashby, Yingjun Guan, Zhe-Sheng Chen

**Affiliations:** ^1^Department of Histology and Embryology, Weifang Medical University, Weifang, China; ^2^Department of Pharmaceutical Sciences, College of Pharmacy and Health Sciences, St. John’s University, Queens, NY, United States; ^3^Tomas Lindahl Nobel Laureate Laboratory, Research Centre, The Seventh Affiliated Hospital, Sun Yat-sen University, Shenzhen, China

**Keywords:** sapitinib, ABCB1, drug resistance, reversal, colon cancer

## Abstract

The efficacy of anti-cancer drugs in patients can be attenuated by the development of multi-drug resistance (MDR) due to ATP-binding cassette (ABC) transporters overexpression. In this *in vitro* study, we determined the reversal efficacy of the epidermal growth factor receptor (EFGR) inhibitor, saptinib, in SW620 and SW720/Ad300 colon cancer cells and HEK293/ABCB1 cells which overexpress the ABCB1 transporter. Sapitinib significantly increased the efficacy of paclitaxel and doxorubicin in ABCB1 overexpressing cells without altering the expression or the subcellular location of the ABCB1 transporter. Sapitinib significantly increased the accumulation of [^3^H]-paclitaxel in SW620/AD300 cells probably by stimulating ATPase activity which could competitively inhibit the uptake of [^3^H]-paclitaxel. Furthermore, sapitinib inhibited the growth of resistant multicellular tumor spheroids (MCTS). The docking study indicated that sapitinib interacted with the efflux site of ABCB1 transporter by π-π interaction and two hydrogen bonds. In conclusion, our study suggests that sapitinib surmounts MDR mediated by ABCB1 transporter in cancer cells.

## Introduction

The efficacy of anticancer drugs, whether in patients 1) who are drug-naïve, 2) have advanced cancer, or 3) that experience treatment relapse, can be attenuated or abrogated by inherent or acquired resistance (1, 2). Furthermore, multi-drug resistance (MDR), defined as the resistance to multiple anticancer drugs that have distinct structures and different mechanism of action, can be extremely difficult to surmount in certain types of cancers (3, 4). There are a number of mechanisms that can produce MDR (2, 5, 6) and one of important mediators is the overexpression of ATP-binding cassette (ABC) transporters (7). The ABC transporter are composed of 49 members and can be categorized into 7 subfamilies, from ABCA to ABCG, all of which function to transport endogenous and xenobiotics across the cell membrane (8). Basically, there are two key structures in ABC transporters, two nucleotide-binding domains (NBDs), where ATP binds and is hydrolyzed, and two trans-membrane binding domains (TMD) where they transport their substrates out of cells or tissues (9, 10). Typically, the amino acid sequence and topology of TMD are not conserved among the various types of ABC transporters (10). The shared structural component is the NBDs, which couple ATP binding and hydrolysis to the TMDs, producing significant conformational changes that ultimately transport substrate molecules from the cells (10). The ABC transporters can efflux a wide variety of endogenous and exogenous molecules, including amino acids, vitamins, lipids, sugars, peptides and certain proteins (11). Furthermore, ABC transporters protect cells against xenobiotics, including certain anti-cancer drugs (12). ABCB1, also known as P-glycoprotein (P-gp), was the first discovered ABC transporter and was shown to play a significant role in regulating colchicine resistance in Chinese hamster ovary cells (13). Numerous studies indicate that the ABCB1 transporter protein is overexpressed in a number of different MDR cancers, producing a significant decrease in the intracellular levels, and thus the efficacy of many different anti-cancer drugs, such as paclitaxel, doxorubicin (14, 15), and targeted therapies such as crizotinib and ceritinib (16). Furthermore, it has been shown that MDR in cancer cells due to the overexpression of the ABCB1 transporter can be surmounted by 1) the blockade of the efflux function of the ABCB1 transporter by compounds such as verapamil, dacomitinib, selonsertib, and other tyrosine kinase inhibitors (17, 18); 2) a decrease in the expression level of the ABCB1 protein or 3) a decrease in the localization of ABCB1 transporter in the cellular membrane (19–21). Thus, the ABCB1 transporter represents a potential therapeutic target for the treatment of MDR in cancers.

**Figure 1:**
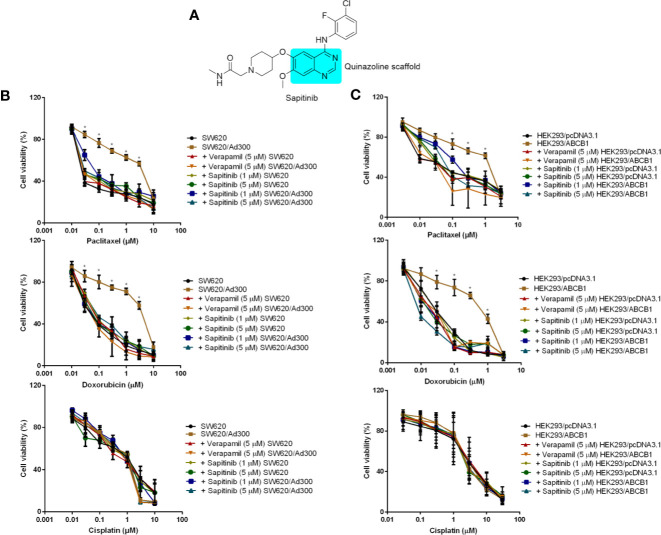
The *in vitro* cytotoxicity of different compounds in ABCB1-overexpressing cells and their corresponding parental cell. **(A)** The structure of sapitinib. **(B)** Cell availability of different treatment on SW620 and SW620/Ad300 cells. **(C)** Cell availability of different treatment on HEK293/pcDNA3.1 and HEK293/ABCB1 cells. *p < 0.05 versus no inhibitor group.

The compound, sapitinib (AZD8931; **Figure 1A**), whose structure contains a quinazoline moiety, is an inhibitor of the epidermal growth factor receptor (EGFR), ErbB2 (HER2), and ErbB3 proteins (22, 23). Sapitinib has *in vitro* and *in vivo* anticancer efficacy in 1) breast cancer cells that overexpress ErbB2; 2) squamous cell carcinoma of the head and neck cell lines; 3) non-small lung cell carcinoma cell lines; and 4) human tumor xenograft models (23, 24). Previously, it has been shown the EGFR inhibitors lapatinib (EGFR), erlotinib (EGFR), afatinib (ErbB1, 2 and 4), gefitinib (EGFR) and dacomitinib (ErbB1-4), all of which have a quinazoline scaffold like sapitinib, are orally efficacious for the treatment of certain types of human cancers (25). Interestingly, studies also indicate that the aforementioned EGFR inhibitors may surmount MDR mediated by ABC transporters in cancers. For example, it has been reported that lapatinib, erlotinib, gefitinib and dacomitinib reversed MDR profiles *in vitro* and *in vivo* by inhibiting the efflux activity of ABCB1 and ABCG2 (18, 26–28), whereas afatinib circumvented MDR mediated by ABCG2 by inhibiting the activity and expression of ABCG2 *in vitro* and *in vivo* (29). In this study, we conducted *in vitro* experiments to determine if sapitinib reverses MDR to paclitaxel and doxorubicin in the human colon cancer cell line, SW620/Ad300, which overexpresses the ABCB1 transporter and in HEK293 cells transfected with the *ABCB1* gene which overexpress the ABCB1 transporter. We also performed experiments to determine the mechanism(s) by which sapitinib overcomes MDR.

## Materials and Methods

### Reagents

Sapitinib was purchased from Selleckchem (Houston, TX). Other chemicals, including 3-(4,5-dimethylthiazol-yl)-2,5-diphenyltetrazolium bromide (MTT), Triton X-100, 4’,6-diamidino-2-phenylindole (DAPI), paclitaxel, doxorubicin, cisplatin, and verapamil, were purchased from Sigma-Aldrich (St. Louis, MO). ^3^H-paclitaxel (15 Ci/mmol) was purchased from Moravek Biochemicals, Inc. (Brea, CA). Dulbecco’s modified Eagle’s Medium (DMEM), fetal bovine serum (FBS), penicillin/streptomycin, and trypsin 0.25% were purchased from Hyclone (GE Healthcare Life Science, Pittsburgh, PA). Antibodies for ABCB1 (Monoclonal, C219) GAPDH (Monoclonal, MA5-15738) and Alexa Fluor 488 conjugated goat anti-mouse IgG secondary antibody was purchased from Thermo Fisher Scientific Inc. (Rockford, IL).

### Cell Lines and Culture

The human colorectal cancer cell lines, SW620 (parental cell line) and SW620/Ad300 cells (which overexpress ABCB1 and are resistant to paclitaxel and doxorubicin), were generously provided by Drs. Susan E. Bates and Robert W. Robey (NIH, Bethesda, MD). The HEK293/pcDNA3.1 and HEK293/ABCB1 cells lines were established by transfecting HEK293 cells with either the empty pcDNA3.1 vector (HEK293/pcDNA3.1) or the vector containing the full length ABCB1 DNA (HEK293/ABCB1), and were cultured in a medium containing 2 mg/mL of G418 2 weeks before conducting the experiments (30). All cell lines used in this study were within 15 to 20 passages from thawing, and were cultured at 37°C/5% CO_2_ with DMEM containing 10% FBS and 1% penicillin/streptomycin.

### Cytotoxicity and Reversal Experiments

First, the cytotoxic effect of sapitinib in the two pairs of cell lines was determined using the MTT assay to determine the non-toxic concentrations that would be used for the remaining experiments. Cells were incubated with sapitinib (0.1–100 µM) and the concentration at which approximately 80% of the cells survived were selected for the drug combination experiments. Second, the IC_50_ values of the anticancer drugs, paclitaxel and doxorubicin, were obtained in the presence or absence of sapitinib. The experiments were repeated at least three times and the standard deviation (SD) values were calculated. The drug verapamil (5 µM), an inhibitor of the ABCB1 transporter, was used as a positive control and cisplatin, which is not an inhibitor or substrate for the ABCB1 transporter, was used as a negative control (31).

### [^3^H]-Paclitaxel Accumulation and Efflux Assay

^3^H-paclitaxel was used to determine the effect of sapitinib on the efflux activity of ABCB1. The accumulation of [^3^H]-paclitaxel in SW620 and SW620/Ad300 cells, in the presence or absence of sapitinib for 2 h, was determined using a Packard TRI-CARB 1900CA liquid scintillation analyzer (Packard Instrument Company, Inc., Downers Grove, IL). Verapamil (5 μM) was used as a positive control inhibitor of ABCB1 (32).

### Western Blot Analysis

Following the incubation with sapitinib at different time intervals, SW620/Ad300 cells were collected and placed in a protein lysis buffer as previously described (33). After the quantification of the lysates by using the bicinchoninic acid (BCA™)-based protein assay (Thermo Scientific, Rockford, IL), a protein lysate (30 μg), mixed with the loading buffer (1/3 of total volume), was pipetted in each lane of a sodium dodecyl sulfate polyacrylamide gel electrophoresis apparatus (SDS-PAGE). An ABCB1 monoclonal antibody, C219 (dilution 1:1,000), and a GAPDH antibody (dilution 1:1,000) were used to identify the presence of ABCB1 and GAPDH, respectively. The protein-antibody complex of ABCB1 and GAPDH was detected using a chemiluminescence detection system (Thermo Scientific, Rockford, IL), and the results were quantified using Image J software (NIH, Bethesda, MD) (18).

### Immunofluorescence Assay

Immunofluorescence analysis was performed to determine the effect of sapitinib on the cellular localization of ABCB1. ABCB1-overexpressing SW620/Ad300 cells (1×10^4^/well in 24-well plate) were incubated with sapitinib (5 µM for 0, 24, 48, and 72 h), and then the cells were fixed using 4% paraformaldehyde at 37°C for 5 min and permeabilized using 0.1% Triton X-100, at 37°C for 5 min. Subsequently, the cells were blocked using 6% BSA at 37°C for 1 h, and co-cultured with the monoclonal antibody C219 at 4°C overnight. The secondary antibody, Alexa Fluor 488 (dilution 1:1,000 in 6% bovine serum albumin in PBS), was added to co-culture with the cells and incubated at 37°C for 1 h. After washing with ice-cold PBS (1 mL) three times, the nuclei were counterstained with 4’,6-diamidino-2-phenylindole (DAPI; 1 μg/mL in PBS) at 37°C for 10 min). An EVOS^®^ FL Auto ﬂuorescence microscope (Life Technologies Corporation, Gaithersburg, MD) was used to detect and capture the immunoﬂuorescence images (34).

### ATPase Assay

The PREDEASY ATPase Kit (TEBU-BIO nv, Boechout, Belgium) was used to determine the vanadate-sensitive ATPase activity of the ABCB1 transporter in membrane vesicles of High Five insect cells, as previously described (35, 36), in the absence and presence of sapitinib (0.625–40 µM).

### Multicellular Tumor Spheroids Assay

Two hundred microliters of culture media, containing 10,000 diluted SW620/Ad300 cells, was added into 0.95% agarose-coated transparent 96-well plates. The cells grew multicellular tumor spheroids (MCTS) aggregates about 400 μm in diameter after 2 days. Subsequently, the MCTSs were incubated with drugs (5 μM of sapitinib and 1 μM of paclitaxel) by replacing 100 μL of the media. All the culture media was refreshed every two days. MCTSs were imaged every day with a phase contrast microscope in a 10X objective to determine their integrity and diameter. The volume of the MCTSs was calculate as: 4/3 × π × (radius)^3^ (37).

### Molecular Modeling Study

The interaction between sapitinib and the drug-binding pocket of ABCB1 (human ABCB1 homology model) was determined using Schrodinger 2018-1 software (Schrödinger, LLC., Cambridge, MA) (38). The ligand and protein preparation were conducted using default protocols as previously described (39). The human ABCB1 homology model was established using the human ABCB1 protein (PDB: 6QEX) that was obtained through the co-crystallization of the human ABCB1 protein docked with paclitaxel (40). The docking grid (length: 25 Å) for the human ABCB1 protein was refined by setting the centroid with the amino acid residues that have been shown to interact with ABCB1 substrates (41). The Glide v7.4 XP (extra precision) docking default protocol (Schrödinger, LLC., Cambridge, MA) was adopted and the induced-fit docking (IFD) was conducted using the best scored binding pose of the ligand and protein from the Glide XP docking to obtain an optimal binding simulation. The top scoring docked pose of ligand-protein complex was subjected to graphical analysis. The docking scores were calculated and expressed as kcal/mol.

### Statistical Analysis

All data were expressed as the means ± SD from at least three repeated experiments. The data were analyzed using a one-way or two-way ANOVA test by GraphPad Prism 7.00 software (GraphPad Software, Inc., San Diego, CA). The *a priori* significance level was P < 0.05.

## Results

### Sapitinib Increased the Efficacy of the ABCB1 Substrates, Paclitaxel and Doxorubicin, in SW620/Ad300 and HEK293/ABCB1 Cells Overexpressing the ABCB1 Transporter

First, we determined the cytotoxicity of sapitinib in SW620/Ad300 (IC_50_ = 35.5 μM) and HEK293/ABCB1 (IC_50_ = 25.5 μM) cells overexpressing the ABCB1 transporter and their parental cell lines, SW620 (IC_50_ = 39.7 μM), and HEK293/pcDNA3.1 (IC_50_ = 33.3 μM), respectively, using the MTT assay. The results of these experiments indicated that the cell viability (**Figure S1**) was at least 80% for sapitinib at concentrations of 1 and 5 μM.

Second, we determined the effect of sapitinib on the efficacy of paclitaxel and doxorubicin in the parental and ABCB1 overexpressing cells (**Figures 1B, C** and **Table 1**). The growth of the parental cell lines, SW620 and HEK293/pcDNA3.1, which expresses very low levels of the ABCB1 transporter, was significantly inhibited by low concentrations of paclitaxel and doxorubicin (**Table 1**). In contrast, in SW620/Ad300 and HEK293/ABCB1 cells, the cytotoxic efficacy of paclitaxel and doxorubicin was significantly decreased due to the overexpression of ABCB1 transporter. In SW620 and HEK293/pcDNA3.1 cells, neither sapitinib nor verapamil (an ABCB1 transport inhibitor) significantly altered the IC_50_ values for paclitaxel and doxorubicin. However, the incubation of SW620/Ad300 and HEK293/ABCB1 cells with sapitinib (1 or 5 μM) significantly increased the cytotoxic efficacy of paclitaxel and doxorubicin, as did the positive control inhibitor, verapamil (**Table 1**). In contrast, neither sapitinib nor verapamil significantly altered the IC_50_ cytotoxic value for cisplatin, which is not a substrate for the ABCB1 transporter (42). In addition, sapatinib, in SW620 and SW620/Ad300 cell lines, reversed resistance to a similar magnitude as the ABCB1 transporter substrate, irinotecan, an anticancer drug used to treat colon cancer, whereas oxaliplatin, which is not an ABCB1 substrate, did not produce a significant effect (**Table S1**). The sensitizing effects were further confirmed in the colon cancer cell line, HCT-15 (**Table S2**).

**Table 1:** Sapitinib sensitized both paclitaxel and doxorubicin to SW620/Ad300 and HEK293/ABCB1 cells.

Treatment	IC_50_ ± SD^a^ (RF^b^) (μM)			
	SW620	SW620/Ad300	HEK293/pcDNA3.1	HEK293/ABCB1
Paclitaxel + Sapitinib (1 μM) + Sapitinib (5 μM) + Verapamil (5 μM)	0.028 ± 0.007 (1.00) 0.029 ± 0.003 (1.04) 0.024 ± 0.005 (0.86) 0.027 ± 0.007 (0.96)	4.705 ± 0.089 (168.03) 0.359 ± 0.066 (12.82)^*^ 0.029 ± 0.005 (1.04)^*^ 0.022 ± 0.004 (0.79)^*^	0.075 ± 0.006 (1.00) 0.065 ± 0.050 (0.87) 0.063 ± 0.009 (0.84) 0.074 ± 0.017 (0.99)	2.024 ± 0.175 (26.99) 0.140 ± 0.108 (1.87)^*^ 0.081 ± 0.021 (1.08)^*^ 0.055 ± 0.014 (0.73)^*^
Doxorubicin + Sapitinib (1 μM) + Sapitinib (5 μM) + Verapamil (5 μM)	0.055 ± 0.039 (1.00) 0.056 ± 0.022 (0.91) 0.044 ± 0.014 (0.79) 0.051 ± 0.022 (0.91)	6.013 ± 0.222 (109.32) 0.060 ± 0.018 (1.07)^*^ 0.083 ± 0.031 (1.48)^*^ 0.054 ± 0.048 (0.96)^*^	0.036 ± 0.011 (1.00) 0.030 ± 0.012 (0.83) 0.022 ± 0.007 (0.61) 0.038 ± 0.003 (1.06)	0.516 ± 0.038 (14.33) 0.037 ± 0.007 (1.03)^*^ 0.021 ± 0.002 (0.58)^*^ 0.034 ± 0.006 (0.94)^*^
Cisplatin + Sapitinib (1 μM) + Sapitinib (5 μM) + Verapamil (5 μM)	1.036 ± 0.226 (1.00) 0.966 ± 0.157 (0.93) 1.004 ± 0.115 (0.97) 0.809 ± 0.350 (0.78)	1.391 ± 0.059 (1.34) 0.915 ± 0.100 (0.88) 1.085 ± 0.115 (1.05) 0.981 ± 0.120 (0.95)	1.293 ± 1.026 (1.00) 1.585 ± 1.030 (1.23) 1.477 ± 0.761 (1.14) 1.476 ± 1.015 (1.14)	1.333 ± 0.807 (1.03) 1.365 ± 0.725 (1.06) 1.287 ± 1.169 (0.99) 1.557 ± 1.099 (1.20)

^*^P < 0.05 versus no inhibitor group.

a Calculated by three independent experiments.

b Calculated by dividing the IC_50_ values of substrates in the presence or absence of sapitinib by the IC_50_ values of parental cells without verapamil or sapitinib.

### Sapitinib Significantly Increased the Intracellular Accumulation of [^3^H]-Paclitaxel in SW620/Ad300 Cancer Cells Overexpressing ABCB1

**Figure 2:**
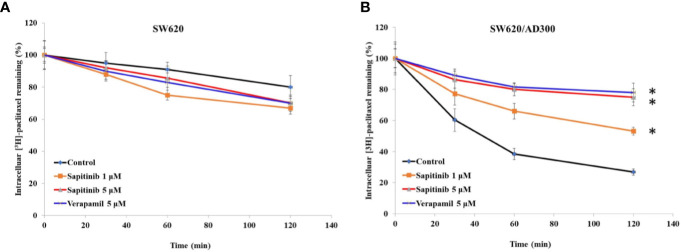
The effect of sapitinib on the *in vitro* intracellular accumulation of [^3^H]-paclitaxel in SW620 and SW620/Ad300 cancer cells. **(A)** The intracellular accumulation of [^3^H]-paclitaxel in SW620 cancer cells following incubation with sapitinib or verapamil. **(B)** The intracellular accumulation of [^3^H]-paclitaxel in SW620/Ad300 cancer cells following incubation with sapitinib and verapamil. The data represent the mean ± SD. *P < 0.05 compared to the control group.

Based on the above results, the *in vitro* efficacy of paclitaxel and doxorubicin in SW620/Ad300 and HEK293/ABCB1 cells was significantly decreased due to an overexpression of the ABCB1 transporter. Consequently, we determined the effect of sapitinib on the efflux function by measuring the accumulation of [^3^H]-paclitaxel in the SW620/Ad300 cancer cells that overexpress the ABCB1 transporter. As shown in **Figure 2**, the intracellular levels of [^3^H]-paclitaxel (approximately 80% remaining after 2 h of incubation with vehicle) in SW620 cells was significantly greater that of SW620/Ad300 cancer cells (26.8% remaining after 2 h of incubation with vehicle). In contrast, the incubation of SW620/Ad300 cancer cells with 1 or 5 μM of sapitinib significantly increased the intracellular accumulation of [^3^H]-paclitaxel to 53.1 and 75%, respectively, compared to cells incubated with vehicle. Furthermore, 5 μM of sapitinib produce a significantly greater increase in intracellular [^3^H]-paclitaxel compared to 1 μM of sapitinib (75.0% *vs* 53.1%). The ABCB1 transporter inhibitor, verapamil, at 5 μM, produced a significantly greater increase in the intracellular levels of [^3^H]-paclitaxel compared to cells incubated with vehicle or 1 μM of sapitinib (**Figure 2**). The verapamil-induced increase in intraceullar levels of [^3^H]-paclitaxel was not significantly different from that of 5 μM of sapitinib.

### Sapitinib Did Not Significantly Alter Either the Expression or the Cellular Localization of the ABCB1 Transporter

**Figure 3:**
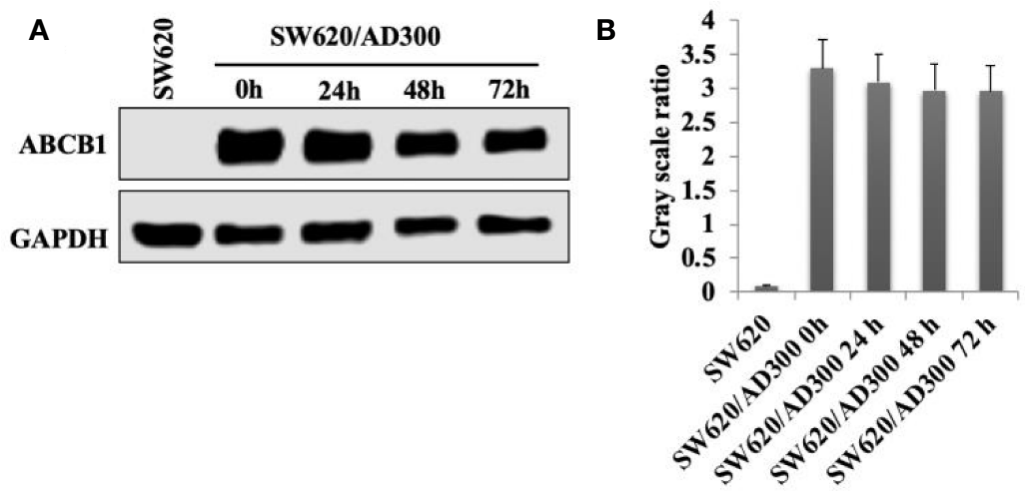
The effect of the incubation of sapitinib on ABCB1 expression in SW620/Ad300 cancer cells. **(A)** Western blots. **(B)** Quantification of the Western blot bands.

As shown in **Figure 3**, the incubation of SW620/Ad300 cells (which overexpress the ABCB1 transporter) with sapitinib (5 μM for 72 h) did not significantly alter the expression of the ABCB1 transporter compared to cells incubated with vehicle. Thus, it is unlikely that saptinib reverses the drug resistance of ABCB1 overexpressing cancer cells by altering the expression of the ABCB1 protein.

### Sapitinib Stimulated the ATPase Activity of ABCB1 and Inhibited the Growth of SW620/Ad300 Multicellular Tumor Spheroids (MCTS)

We next conducted experiments to ascertain if sapitinib alters the ATPase activity of the ABCB1 transporter. Our results, as shown in **Figure 5A**, indicated that sapitinib (0–40 μM) produced a 4.2-fold increase in the basal level of ATPase activity compared to cells incubated with vehicle. Sapitinib, at 5.3 μM, increased ATPase activity by 50%.

**Figure 4:**
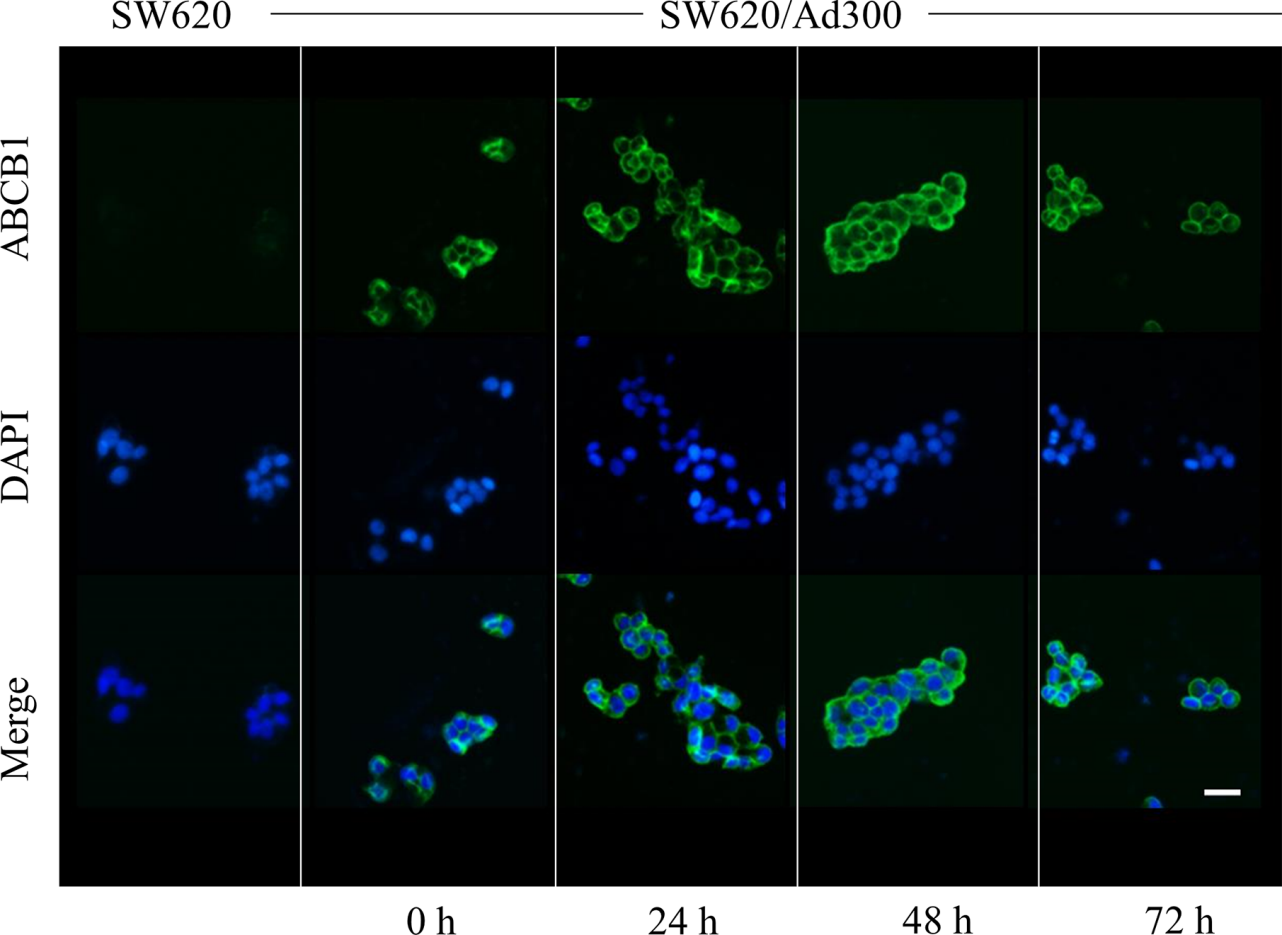
The effect of sapitinib on the cellular location of ABCB1 in SW620/Ad300 cancer cells. ABCB1-overexpressing SW620/Ad300 cells were seeded in 24-well plate and incubated with or without sapitinib (5 µM) for 0, 24, 48 and 72 h. The cells were then fixed using 4% paraformaldehyde at 37°C for 5 min. Subsequently, the cells were blocked using 6% BSA at 37°C for 1 h and co-cultured with the monoclonal antibody C219 at 4°C overnight. The secondary antibody, Alexa Fluor 488 was added to co-culture with the cells and incubated at 37°C for 1 h. After washing with ice-cold PBS, the nuclei were counterstained with DAPI at 37°C for 10 min. An EVOS^®^ FL Auto ﬂuorescence microscope was used to detect and capture the immunoﬂuorescence images. SW620 cells were used as a negative control.

It is possible that sapitinib could increase the efficacy of paclitaxel and doxorubicin by decreasing the number of ABCB1 transporter in the cell membrane, thereby decreasing the efflux of these drugs. As shown in **Figure 4**, ABCB1 transporter in the cells incubated with vehicle were primarily localized in the cell membrane (as shown in green). The incubation of cells with sapitinib (5 μM for 72 h) did not significantly alter the cellular localization of the ABCB1 protein as compared to cells incubated with vehicle.

**Figure 5:**
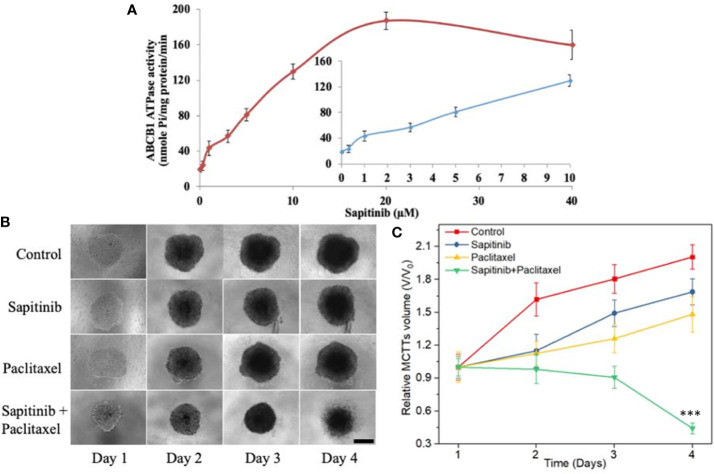
The effect of sapitinib on the ATPase activity of ABCB1 transporter and the growth of multicellular tumor spheroids. **(A)** Effect of sapitinib (0.625–40 μM) on the ATPase activity of ABCB1 transporter in membrane vesicles of High Five insect cells. **(B)** The effect of the incubation of sapitinib, paclitaxel and the paclitaxel + sapitinib on the growth of SW620/Ad300 MCTSs (sapitinib: 5 μM; paclitaxel: 1 μM). Scale bar = 300 μm. **(C)** The corresponding relative volume change (V/V0) from control ***P < 0.001 versus Paclitaxel.

We developed MCTSs of SW620/Ad300 cells to further confirm the reversal efficacy of sapitinib for the ABCB1 transporter. As shown in **Figures 5B, C**, there was a significant increase in the size of the SW620/Ad300 MCTSs after incubation with sapitinib (5 μM) or paclitaxel (1 μM) alone, indicating that the sapitinib was not cytotoxic to SW620/Ad300 cells and these cells are resistant to paclitaxel. However, preincubaiton with sapitinib, followed by incubation with paclitaxel, significantly inhibited the growth of SW620/Ad300 MCTSs compared to cells incubated with sapitinib or paclitaxel.

### Induced-Fit Docking (IFD) Simulation Interactions Between ABCB1 and Sapitinib

**Figure 6:**
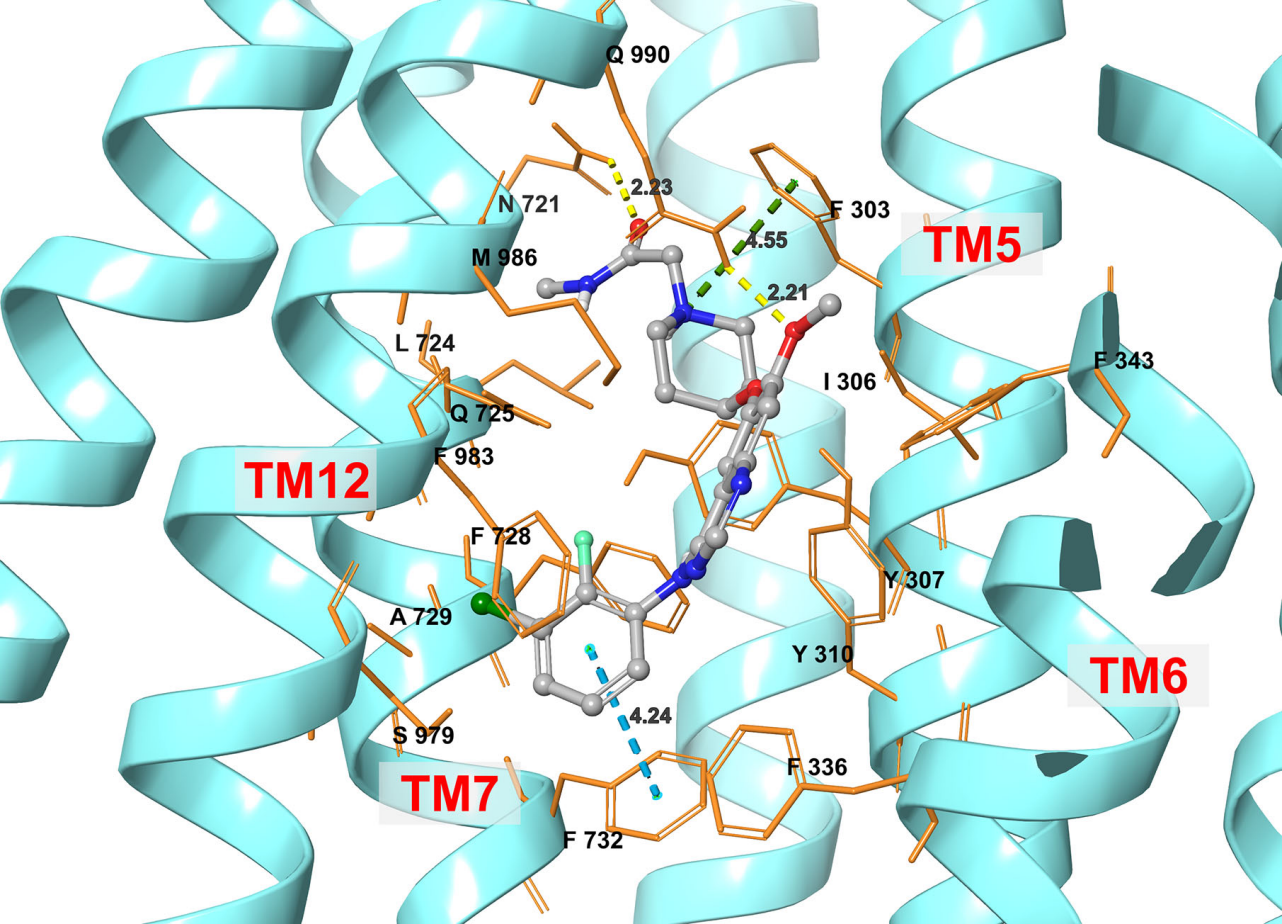
An illustration of the interaction between sapitinib and the human ABCB1 transporter protein based on docking analysis.

The best docking pose for sapitinib at the human homology ABCB1 protein (IFD Glide score: -11.655 kcal/mol) is shown in **Figure 6**. Sapitinib was predicted to interact into the drug-substrate binding site of the human ABCB1 protein, which is lined by a number of hydrophobic and aromatic residues that belong to transmembrane helices 5, 6, 7, and 12 of ABCB1 TMD, including F303, I306, Y307, Y310, F336, F343, L724, F728, A729, and F732 (**Figure 6**). The docking analysis indicated that sapitinib had hydrogen-bonding interactions with N721 and Q990. The anilino ring of sapitinib was involved in a π-π interaction with the phenyl ring of F732. Finally, the amide nitrogen in the piperidine ring of sapitinib was predicted to interact with the phenyl ring of F303 *via* π-cation interaction.

## Discussion

Under physiological conditions, ABCB1 is expressed at relatively low levels in the parent colon cancer cells compared to those in the drug resistant cell lines (43). SW620/Ad300 cells, which were used in this study, are a doxorubicin-selected cell line that was incubated with various concentrations of doxorubicin over a period of six months. The expression of the ABCB1 transporter has been reported to vary in colon cancer patients depending upon the chemotherapeutic regimen (44–46). In patients treated with 5-fluorouracil (5-FU), the ABCB1 transporter was reported to be slightly overexpressed in cancer tissue compared with normal tissue (45), whereas the ABCB1 transporter was significantly overexpressed in patients treated with irinotecan (46). It has been reported that polymorphisms in the ABCB1 transporter gene may contribute to the progression of colon cancer (44). Among certain populations, there is a correlation between ABCB1 gene polymorphisms and the risk for colorectal cancer (47), although it has also been shown that certain ABCB1 variants. such as C3435T, T129C and G2677T, were not correlated with an increased risk of colorectal cancer (48, 49). Overall, the overexpression of the ABCB1 transporter can produce drug resistance in various types of cancer, including colon cancer, although additional research is needed.

In the current study, we conducted experiments to determine if sapitinib could increase the efficacy (i.e., resensitize) of the ABCB1 substrates, paclitaxel and doxorubicin, in SW620 and the ABCB1-overexpressing SW620/Ad300 cells, as well as HEK293 and ABCB1 transfected HEK293/ABCB1 cells. HEK293/ABCB1 cells were used to determine if the reversal efficacy of sapatinib was mediated by its effects on the ABCB1 transporter, as opposed to other mechanisms, as unlike SW620/Ad300 cells, where the resistance to certain drugs is mediated by a number of mechanisms, the resistance of HEK293/ABCB1 cells to paclitaxel and doxorubicin but not to platinum-based anticancer drugs, is due only to the overexpression of the ABCB1 transporter.

One of the main findings of this study was that *in vitro*, sapitinib significantly increased the efficacy of the anti-cancer drugs paclitaxel and doxorubicin in SW620/Ad300 colon cancer cells and ABCB1 transfected HEK293/ABCB1 cells, which overexpress ABCB1 transporter. Indeed, the IC_50_ values for paclitaxel and doxorubicin, which are ABCB1 substrates, were significantly higher in the ABCB1 overexpressing cells compared to the parental cell lines. In fact, the resistance to paclitaxel and doxorubicin was completely reversed by either 1 or 5 μM of sapitinib, in the HEK293/pcDNA3.1 and cancer cells overexpressing ABCB1 transporter. Furthermore, these concentrations of sapitinib alone did not significantly alter cell viability. As previously reported, verapamil (5 μM), an inhibitor of the ABCB1 transporter (50), completely reversed the drug resistance of the ABCB1 overexpressing SW620/Ad300 colon and transfected HEK293/ABCB1 cells (18, 26, 27, 51). It is unlikely that the antagonism of EGFR receptors is contributing to the cytotoxicity of sapitinib as it has been reported that there is no difference in EGFR receptor expression between the non-resistant parental and ABCB1 overexpressing resistant cell lines. Furthermore, it is likely that the effects of quinazoline-based EGFR inhibitors, including sapitinib, on ABC transporters, are due to their structures, indicating that certain structures inhibit the efflux function of ABC transporters. However, we cannot rule out the possibility that the increase in anticancer drug efficacy produced by sapitinib could be due to its inhibition of tyrosine kinases (52, 53). Finally, sapitinib did not significantly alter the cytotoxic efficacy of cisplatin and oxaliplatin, two anti-cancer drugs that are not substrates for the ABCB1 transporter (42). Thus, sapitinib, at concentrations of 1 or 5 μM, reverses ABCB1-mediated resistance to paclitaxel by affecting the function but not the expression of the ABCB1 transporter.

Another major finding of this study was that, *in vitro*, sapitinib (1 or 5 μM) significantly increased the intracellular accumulation of [^3^H]-paclitaxel in SW620/Ad300 cells overexpressing the ABCB1 transporter compared to cells incubated with vehicle. In contrast, sapitinib did not significantly alter the intracellular accumulation of [^3^H]-paclitaxel in the parental SW620 cell, which do not overexpress the ABCB1 transporter. Our findings suggest that sapitinib antagonizes the efflux function of the ABCB1 transporter, thereby attenuating the efflux of ^3^H-paclitaxel, and potentially, other anticancer drugs that are ABCB1 substrates, thus increasing the intracellular drug concentrations, leading to a restoration of drug efficacy in ABCB1 overexpressing cells. Additional experiments, such as determining the effect of sapitinib on the binding of radioactive ligand, ^125^I-IAAP, which is substrate for the ABCB1 transporter (54, 55), could provide additional insight into its mechanism.

It is possible that sapitinib could decrease the intracellular accumulation of doxorubicin and paclitaxel by decreasing the expression level of the ABCB1 transporter protein. However, Western blot data indicated that the incubation of SW620/Ad300 cancer cells with 5 μM of sapitinib for 72 h did not significantly alter the levels of the ABCB1 transporter protein compared to cells incubated with vehicle. Thus, under the experimental conditions in this study, it is unlikely that sapitinib’s potentiation of the efficacy of paclitaxel and doxorubicin is due a decrease in the expression of the ABCB1 transporter protein. However, it is possible that incubation period > 72 h could significantly alter ABCB1 protein expression, although this remains to be delineated. The sapitinib-induced reversal of MDR to paclitaxel and doxorubicin in SW620/Ad300 cancer cells could also result from a decrease *in vitro* in the number or density of ABCB1 transporter present in the cancer cell membrane. However, this is unlikely as the incubation of SW620/Ad300 cancer cells with 5 μM of sapitinib for 72 h did not significantly alter the subcellular distribution of the ABCB1 transporter compared to cells incubated with vehicle. Again, as with the ABCB1 protein expression experiments, future experiments must be conducted to determine if longer incubation times affect the subcellular distribution of the ABCB1 transporter.

It has been shown that the drug efflux function of the ABCB1 transporter is coupled to ATP hydrolysis, which is increased by ABCB1 substrates (56, 57). Therefore, we determined the effect of sapitinib on the hydrolysis of ATP by the ATPase domain of the ABCB1 transporter. The incubation of membrane vesicles from High Five insect cells containing the ABCB1 protein indicated that sapitinib (0.625-40 μM) produced a concentration-dependent increase in ATPase activity, and the maximal stimulation was 4.2-fold greater than the basal level of ATPase activity. The increase in ABCB1 ATPase activity produced by sapitinib suggests that it may competitively limit the uptake of substrate by the ABCB1 transporter by interacting with the TMD drug-substrate binding site. Indeed, our docking studies indicated that sapitinib interact with the drug-substrate binding site in the human homology model of the ABCB1 transporter. Sapitinib formed strong hydrogen bonds, π-π and π-cation interactions with the active center of ABCB1.

Three dimensional MCTSs, compared to 2D cell culture, represents a more relevant model to determine the anticancer efficacy of drugs because it closely mimics the cell-cell interactions and more importantly, the microenvironmental conditions occurring *in vivo* which can significantly affect cell proliferation, differentiation and tumor growth (58). Our results showed that the SW620/Ad300 MCTSs had a clear loss MCTSs matrix after four days, suggesting that SW620/Ad300 cells died, indicating that the combination of sapitinib and paclitaxel may exert similar synergistic effects *in vivo*. These results further indicated that sapitinib significantly increased the efficacy of paclitaxel in resistance mediated by the overexpression of the ABCB1 transporter. Further research is needed to further validate the reversal efficacy of sapitinib in an *in vivo* tumor xenograft model.

In conclusion, the results of this *in vitro* study indicate that sapitinib completely reverses the resistance of SW620/Ad300 and HEK293/ABCB1 cells overexpressing ABCB1 transporter to the anti-cancer drugs, paclitaxel and doxorubicin, which are ABCB1 substrates. It is likely that sapitinib reverses resistance to paclitaxel and doxorubicin by inhibiting the efflux function of ABCB1 as it 1) increases the intracellular accumulation of ^3^H-paclitaxel and 2) increases ABCB1 ATPase activity, thereby blocking the binding of substrate drugs to the ABCB1 transporter, which can inhibit the efflux function. Furthermore, sapitinib does not significantly alter 1) the expression levels of the ABCB1 protein and 2) the subcellular localization of the ABCB1 transporter. Finally, docking data indicates that sapitinib interacts with the drug binding pocket of ABCB1, not the ATP binding pocket. Overall, our results with sapitinib, as well as those obtained with other quinazoline-based EGFR inhibitors, suggest that these compounds may have the potential to treat MDR in certain cancers that overexpress ABCB1 transporter. However, this hypothesis remains to be verified.

## Data Availability Statement

The raw data supporting the conclusions of this article will be made available by the authors, without undue reservation.

## Author Contributions

Conceptualization: Z-SC. Methodology: H-LG, PG, and Z-SC. Investigation: H-LG, PG, QC, YA, Z-XW, LZ, Z-NL, and Q-XT. Writing original draft: H-LG, PG, CA, and QC. Funding acquisition: Z-SC, YG, and H-LG. Resources: YG and Z-SC. Supervision: YG and Z-SC. All authors contributed to the article and approved the submitted version.

## Funding

This work was supported by the Program of Study Abroad for Scholars sponsored by Weifang Medical University to H-LG. This research was partially funded by the Department of Pharmaceutical Sciences, College of Pharmacy and Health Sciences, St. John’s University, Weifang Medical University, and partially by the National Natural Science Foundation of China, grant numbers 81872901 and 81871006.

## Conflict of Interest

The authors declare that the research was conducted in the absence of any commercial or financial relationships that could be construed as a potential conflict of interest.
