# Supplementary material for: Sapitinib Reverses Anticancer Drug Resistance in Colon Cancer Cells Overexpressing the ABCB1 Transporter
Source: Front Oncol. 2020 Oct 9;10:574861. doi: 10.3389/fonc.2020.574861 (PMC7581728; doi:10.3389/fonc.2020.574861)
Supplement: Supplementary file 1 [file DataSheet_1.pdf]

Supporting Information

Sapitinib reverses anticancer drug resistance in colon cancer cells overexpressing the ABCB1 transporter

Hai-Ling Gao[1,2,#], Pranav Gupta[2,#], Qingbin Cui[2], Yunali V. Ashar[2], Zhuo-Xun Wu[2], Leli Zeng[2], Zi-Ning Lei[2], Charles R. Ashby, Jr.[2], Qiu-Xu Teng[2], Dong-Hua Yang[2], Yingjun Guan[1,*], Zhe-Sheng Chen[2,*]

Legends

Figure S1. Cell viability for SW620, SW620/Ad300, HEK293 and HEK293/ABCB1 cells after incubation with sapitinib. The data represent the mean ± SD.

Table S1. The reversal efficacy of Sapitinib in SW620 and SW620/Ad300 cells.

Table S2. The reversal efficacy of Sapitinib in HCT-15 cells.

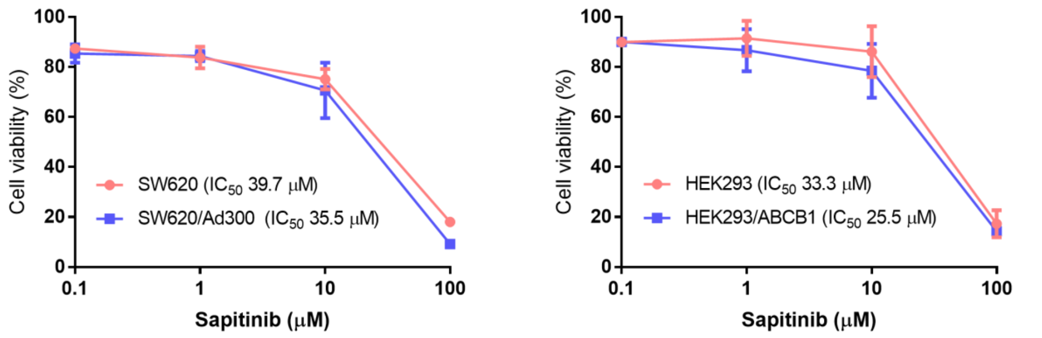

Figure S1: Cell viability for SW620, SW620/Ad300, HEK293, and HEK293/ABCB1 cells after incubation with sapitinib

Table S1

Treatment	IC_{50} value ± SD[a] (μM, Resistance fold[b])	
	SW620	SW620/Ad300
**Irinotecan**	1.05 ± 0.10 (1.00)	82.54 ± 13.76 (78.71)
+ Sapitinib 1 μM	1.20 ± 0.16 (1.14)	4.39 ± 0.73 (4.18) *
+ Sapitinib 5 μM	1.18 ± 0.37 (1.12)	1.49 ± 0.25 (1.42) *
+ Verapamil 5 μM	1.19 ± 0.11 (1.13)	1.99 ± 0.33 (1.90) *
**Oxaliplatin**	13.50 ± 2.25 (1.00)	15.60 ±1.11 (1.16)
+ Sapitinib 1 μM	16.52 ± 2.75 (1.22)	17.09 ±0.81 (1.27)
+ Sapitinib 5 μM	12.53 ± 2.09 (0.93)	13.87 ±0.66 (1.03)
+ Verapamil 5 μM	15.48 ± 2.58 (1.15)	17.92 ±0.85 (1.33)

[a] IC_{50} values are represented as the means ± SD of at least three independent experiments performed in triplicate.

[b] Rf: Resistance - fold was calculated by dividing the IC_{50} values of the substrates in the presence or absence of inhibitor by the IC_{50} of the parental cells in the absence of

the inhibitor.

* $P < 0.05$ versus the control group in the absence of the reversal compound

Table S2

Treatment	IC_{50} value ± SD[a] (μM, Resistance fold[b])
	HCT-15
**Paclitaxel**	0.92 ±0.08 (1.00)
+ Sapitinib 1 μM	0.60 ±0.14 (0.65)
+ Sapitinib 5 μM	0.27 ±0.04 (0.29) *
+ Verapamil 5 μM	0.35 ±0.06 (0.39) *
**Doxorubicin**	2.49 ±0.23 (1.00)
+ Sapitinib 1 μM	2.15 ±0.20 (0.86)
+ Sapitinib 5 μM	0.96 ±0.13 (0.39) *
+ Verapamil 5 μM	0.71 ±0.12 (0.28) *
**Irinotecan**	2.34 ±0.21 (1.00)
+ Sapitinib 1 μM	0.48 ±0.11 (0.20) *
+ Sapitinib 5 μM	0.28 ±0.04 (0.18) *
+ Verapamil 5 μM	0.37 ±0.06 (0.16) *
**Oxaliplatin**	0.88 ±0.08 (1.00)
+ Sapitinib 1 μM	0.95 ±0.22 (1.08)
+ Sapitinib 5 μM	0.94 ±0.12 (1.07)
+ Verapamil 5 μM	0.83 ±0.14 (0.95)

[a] IC_{50} values are represented as the means ± SD of at least three independent experiments performed in triplicate.

[b] Rf: Resistance - fold was calculated by dividing the IC_{50} values of the substrates in

the presence or absence of the inhibitor by the IC_{50} of the parental cells in the absence of the inhibitor.

* $P < 0.05$ versus the control group in the absence of the reversal compound.
